# Increased sCD163 and sCD14 Plasmatic Levels and Depletion of Peripheral Blood Pro-Inflammatory Monocytes, Myeloid and Plasmacytoid Dendritic Cells in Patients With Severe COVID-19 Pneumonia

**DOI:** 10.3389/fimmu.2021.627548

**Published:** 2021-02-26

**Authors:** Maria Antonella Zingaropoli, Parni Nijhawan, Anna Carraro, Patrizia Pasculli, Paola Zuccalà, Valentina Perri, Raffaella Marocco, Blerta Kertusha, Guido Siccardi, Cosmo Del Borgo, Ambrogio Curtolo, Camilla Ajassa, Marco Iannetta, Maria Rosa Ciardi, Claudio Maria Mastroianni, Miriam Lichtner

**Affiliations:** ^1^ Department of Public Health and Infectious Diseases, Sapienza University of Rome, Rome, Italy; ^2^ Infectious Diseases Unit, SM Goretti Hospital, Sapienza University of Rome, Latina, Italy; ^3^ Department of System Medicine, Tor Vergata University of Rome, Rome, Italy

**Keywords:** monocytes, dendritic cells, pDCs, mDCs, SARS-CoV-2, flow cytometry, ELISA

## Abstract

**Background:**

Emerging evidence argues that monocytes, circulating innate immune cells, are principal players in COVID-19 pneumonia. The study aimed to investigate the role of soluble (s)CD163 and sCD14 plasmatic levels in predicting disease severity and characterize peripheral blood monocytes and dendritic cells (DCs), in patients with COVID-19 pneumonia (COVID-19 subjects).

**Methods:**

On admission, in COVID-19 subjects sCD163 and sCD14 plasmatic levels, and peripheral blood monocyte and DC subsets were compared to healthy donors (HDs). According to clinical outcome, COVID-19 subjects were divided into ARDS and non-ARDS groups.

**Results:**

Compared to HDs, COVID-19 subjects showed higher sCD163 (p<0.0001) and sCD14 (p<0.0001) plasmatic levels. We observed higher sCD163 plasmatic levels in the ARDS group compared to the non-ARDS one (p=0.002). The cut-off for sCD163 plasmatic level greater than 2032 ng/ml was predictive of disease severity (AUC: 0.6786, p=0.0022; sensitivity 56.7% [CI: 44.1–68.4] specificity 73.8% [CI: 58.9–84.7]). Positive correlation between plasmatic levels of sCD163, LDH and IL-6 and between plasmatic levels of sCD14, D-dimer and ferritin were found. Compared to HDs, COVID-19 subjects showed lower percentages of non-classical (p=0.0012) and intermediate monocytes (p=0.0447), slanDCs (p<0.0001), myeloid DCs (mDCs, p<0.0001), and plasmacytoid DCs (pDCs, p=0.0014). Compared to the non-ARDS group, the ARDS group showed lower percentages of non-classical monocytes (p=0.0006), mDCs (p=0.0346), and pDCs (p=0.0492).

**Conclusions:**

The increase in sCD163 and sCD14 plasmatic levels, observed on hospital admission in COVID-19 subjects, especially in those who developed ARDS, and the correlations of these monocyte/macrophage activation markers with typical inflammatory markers of COVID-19 pneumonia, underline their potential use to assess the risk of progression of the disease. In an early stage of the disease, the assessment of sCD163 plasmatic levels could have clinical utility in predicting the severity of COVID-19 pneumonia.

## Introduction

The coronavirus disease 2019 (COVID-19) is characterized by diffuse alveolar damage and infiltration of monocytes, macrophages, and lymphocytes in pulmonary interstitium, which interfere with alveolar gas exchange and may lead to acute respiratory distress syndrome (ARDS) (1). An exacerbate inflammatory response to severe acute respiratory syndrome coronavirus-2 (SARS-CoV-2) with the overproduction of many inflammatory cytokines, known as macrophage activation syndrome (MAS) or cytokine storm, is thought to be a major cause of disease severity and death in patients with COVID-19 pneumonia (2). Indeed, the crucial determinant of severe COVID-19 pneumonia appears to be a significant increase in systemic pro-inflammatory cytokines and other inflammatory markers (3–5), indicative of a cytokine storm. This phenomenon corresponds to what is seen in other viral infections and bacterial sepsis (6, 7).

The proliferation and activation of monocytes/macrophages is the most significant step in the initiation of the immunopathogenesis of a wide range of infections (8) and is thought to contribute to the pathogenesis of COVID-19 pneumonia concomitantly with the cytokine storm (9). Although several studies indicate a potential role of monocytes in COVID-19 pneumonia immunopathology, there is a lack of information regarding the role of soluble (s)CD163, which is shed from the corresponding membrane marker of monocyte/macrophage and increases during inflammatory responses (10). CD163 is a receptor that binds haptoglobin-hemoglobin complexes and is mainly expressed on macrophages and monocytes. As a result of the shedding, during inflammation and macrophage activation, the extracellular portion of CD163 circulates in the blood as sCD163 (11). High CD163 expression on alveolar macrophages was reported in patients with chronic obstructive pulmonary disease (COPD) (12) and in idiopathic pulmonary fibrosis (13).

CD14 is a surface protein found on monocytes, macrophages, and activated neutrophils and serves as the cellular receptor for lipopolysaccharide (LPS), which is a major component of the outer membrane of gram-negative bacteria (14). CD14 plays an important role in mounting the host response to bacterial pathogens. CD14 can indirectly trigger the release of interleukin (IL)-1, IL-6, IL-8, and tumor necrosis factor (TNF)-α (15). CD14-dependent mechanisms of inflammation have been identified in ARDS (16), sarcoidosis (17), and asthma (18). During cellular activation, the soluble form of CD14 (sCD14) is generated by proteolytic shedding of the membrane-associated form (mCD14) (17, 18).

Macrophages and monocytes are important contributors to innate and adaptive immune responses. Monocytes are cells of the innate immune system implicated in inflammatory responses, phagocytosis, antigen presentation, and a multiplicity of other immune function processes (19). Moreover, during sterile and non-sterile inflammation, circulating monocytes extravasate into peripheral tissues to differentiate into macrophages or dendritic cells. In humans, monocytes are generally described as consisting of three subsets based on the expression of CD14 and CD16. They are commonly identified as classical (CD14++CD16-), intermediate (CD14++CD16+), and non-classical (CD14+CD16+) subtypes (20). Upon viral infection, monocytes respond with an elevated expression of proinflammatory signaling molecules and antiviral responses, as in the case of infections caused by Influenza, Chikungunya, human herpes, and Zika viruses (21).

Two types of dendritic cells (DCs), with an immature phenotype, can be identified in human blood: myeloid DCs (mDCs) and plasmacytoid DCs (pDCs) (22). mDCs express CD11c and for their growth and survival require granulocyte–macrophage colony-stimulating factor (GM-CSF).These cells are specialized in antigen uptake, T cell activation and secretion of interleukin (IL)-12 and IL-18. pDCs express CD123 and for their survival depend on IL-3. These cells produce high levels of type I interferons (IFNs) in response to viral infections (23, 24). During viral infection, an *ex vivo* significant reduction in circulating DC counts was reported; in particular, a decrease in both mDCs and pDCs was observed in individuals infected with human immunodeficiency virus (HIV) and in those infected with hepatitis C virus (HCV) (25–27). An additional population of myeloid cells sharing functional and phenotypic characteristics with mDCs was identified by Schäkel and colleagues. These cells known as **“**slanDCs**”** selectively express the 6-sulfo LacNAc1 (slan) carbohydrate modification of P-selectin glycoprotein ligand-1 (PSGL-1), which is specifically recognized by the monoclonal antibody M-DC8 (28). These cells are major producers of pro-inflammatory cytokines, such as TNF-α and IL1β and an increase in their frequency and numbers was reported in viremic HIV positive patients (29–32).

The aim of this study was to investigate the plasmatic levels of two blood markers that rise with macrophage/monocyte activation, sCD163 and sCD14, in patients with COVID-19 pneumonia and assess their role in predicting disease severity. Moreover, an immunophenotyping analysis was performed on peripheral whole blood to characterize monocyte and DC subsets in patients with COVID-19 pneumonia.

## Material and Methods

### Study Population and Clinical Parameters

From March 2020 to April 2020, patients with COVID-19 pneumonia (COVID-19 subjects) admitted to the Department of Public Health and Infectious Diseases, Policlinico Umberto I, Sapienza University of Rome and S.M. Goretti Hospital of Latina, were enrolled. COVID-19 related pneumonia was diagnosed by computed tomography (CT scan) of the chest associated with SARS-CoV-2 RNA detection from a nasopharyngeal swab through a commercial reverse transcription polymerase chain reaction (RT-PCR) kit, following manufacturer**’**s instructions (RealStar**^®^** SARS-CoV-2 Altona Diagnostic, Germany).

On hospital admission, clinical information and routine laboratory exams, including demographics, respiratory parameters with arterial oxygen partial pressure**/**fraction of inspired oxygen (PaO2/FiO2) ratio, lactate dehydrogenase (LDH), C-reactive protein (CRP), ferritin, D-dimer, blood lymphocytes counts were collected.

According to clinical outcome, COVID-19 subjects were stratified into two groups: ARDS and non-ARDS. ARDS was defined according to the 2012 Berlin criteria (33). Moreover, patients were further classified according to WHO guidelines into three groups: critical, severe and mild (34). Healthy donors (HDs) were enrolled as control group, with similar age and sex distribution compared to COVID-19 subjects, without any symptom, and with a negative nasopharyngeal swab for SARS-CoV-2 RNA detection and undetectable anti-SARS-CoV-2 specific IgG.

### Quantification of sCD163 and sCD14 Plasmatic Levels

On hospital admission, during routine clinical testing, whole blood was collected in heparin tubes. Plasma was obtained after centrifugation and immediately stored at -80°C until use. sCD163 and sCD14 plasmatic levels were quantified using enzyme-linked immunosorbent assay (ELISA) kits (Quantikine, R&D Systems, Minneapolis, Minnesota, USA) as previously described in details (35). Standard curves and samples were tested in duplicate. The limit of detection for sCD163 and sCD14 was 0.177 ng/ml and 0.125 ng/ml, respectively.

### Flow Cytometry Characterization of Peripheral Blood Monocytes and DCs

**Figure 1:**
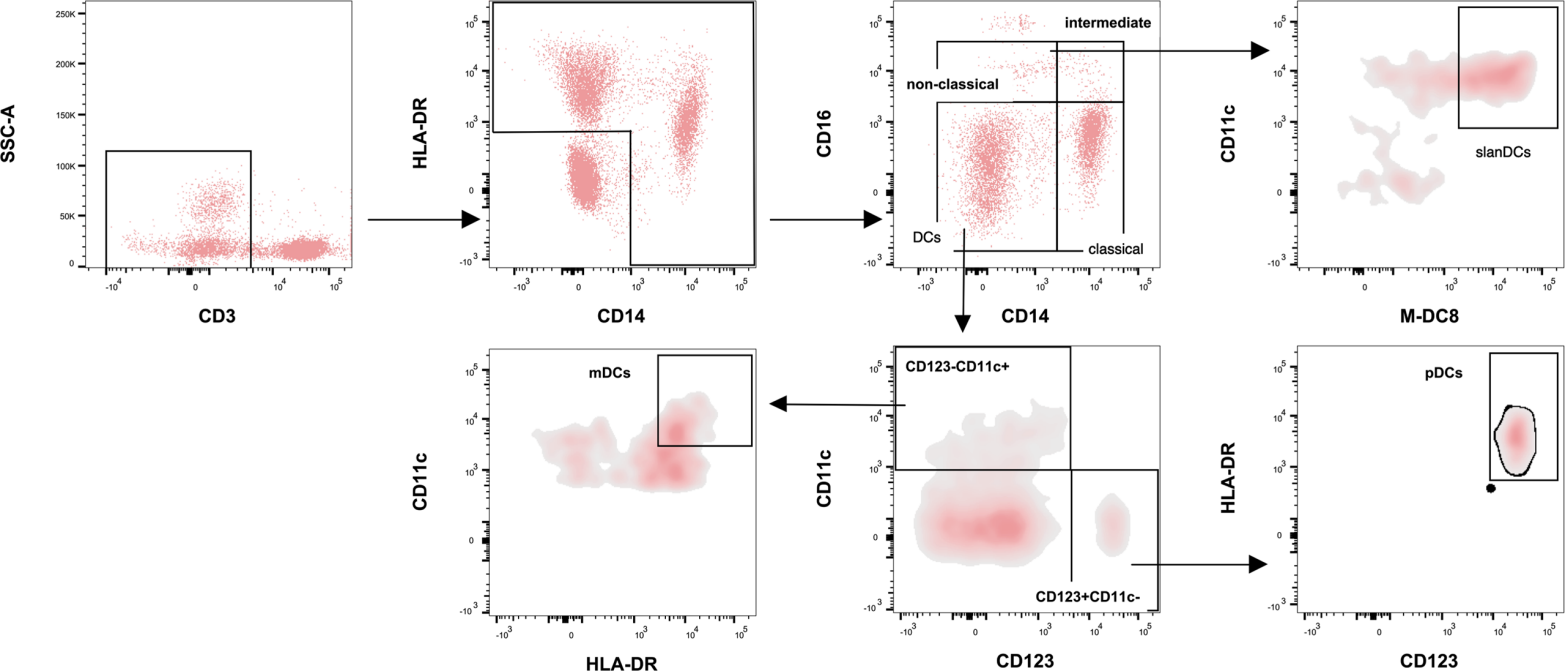
Flow cytometry strategy for monocyte and dendritic cell (DC) subsets. Figure shows monocyte subsets characterization after exclusion of CD3+ and HLA-DR-CD14- cells. According to CD14 and CD16 expression, monocytes were defined as non-classical (CD14+CD16+), intermediate (CD14++CD16+), and classical monocytes (CD14++CD16-). The frequency of the slanDC cells was evaluated into the gate of non-classical monocytes, after gating the cells co-expressing M-DC8 and CD11c. Finally, in the CD14-CD16- gate, DC subsets were characterized according to the expression of CD123 and CD11c as CD123-CD11c+ and CD123+CD11c-. The frequency of mDCs was evaluated in the CD123-CD11c+ gate according to the high expression of CD11c and HLA-DR. The frequency of pDCs was evaluated in the CD123+CD11c- gate according to the high expression of CD123 and HLA-DR.

On hospital admission, for a subgroup of the study population (COVID-19 subjects hospitalized in Rome and HDs) peripheral whole blood samples, collected in heparin tubes, were stained within 3 h from collection. Circulating monocytes and DCs were characterized using multiparametric flow cytometry. The subset distribution and immunophenotype of monocytes and DCs were investigated (**Figure 1**). As previously described (36), Brilliant Violet 510-conjugated anti-CD3, APC-conjugated anti-CD14, PerCp/Cy5.5-conjugated anti-HLA-DR, and PE/Cy7-conjugated anti-CD16 antibodies were used to identify specific monocyte subsets: non-classical monocytes (CD14+CD16+), intermediate monocytes (CD14++CD16+) and classical monocytes (CD14++CD16-). The FITC-conjugated anti-M-DC8 and PE-conjugated anti-CD11c antibodies were used to identify slanDC cells (M-DC8+CD11c+). Finally, the Pacific Blue-conjugated anti-CD123 and PE-conjugated anti-CD11c antibodies were used to identify dendritic cell subsets: myeloid dendritic cells (mDCs, CD123-CD11c+) and plasmacytoid dendritic cells (pDCs, CD123+CD11c-). All the antibodies were from BioLegend, with the exception of M-DC8 and CD11c which were from Miltenyi Biotec.

Briefly, 50 µl of whole blood were stained with the appropriate mix of monoclonal antibodies, incubated in darkness at 4°C for 20 min. After first incubation, red blood cells were lysed using the BD lyse solution, in the darkness at room temperature for 10 min (BD Biosciences). The cells were washed twice in phosphate-buffered saline (PBS) containing 1% of Fetal Calf Serum (FCS). Finally, cells were fixed in PBS containing 0.5% of formaldehyde (Sigma-Aldrich) before analysis. The stained samples were acquired using the MACSQuant Flow Cytometer (Miltenyi Biotec, Germany) and analyzed using FlowJo™ v10.6.2 software.

### Ethics Statement

This study was approved by the Ethics Committee of Policlinico Umberto I, Sapienza University of Rome (protocol number 298/2020) and the Ethic Committee Lazio 2 (protocol number 0080757/2020). Each subject gave written informed consent for participation to the study.

### Statistical Analysis

All statistical analyses were performed using GraphPad Prism v.8.4.1 software and two-tailed p **≤** 0.05 was considered statistically significant. Values are represented as median and interquartile range (IQR). The nonparametric comparative Mann-Whitney test was used for comparing medians between COVID-19 subjects and HDs and between ARDS and non-ARDS groups. The nonparametric Kruskal-Wallis test with Dunn**’**s post-test was used for comparing medians of ARDS and non-ARDS groups with HDs. Spearman rank correlation analysis, which is known to be robust and insensitive to non‐normality of data distribution and outliers, was used to assess the relation between percentages of circulating monocytes and DCs, clinical and laboratory data, and sCD163 and sCD14 plasmatic levels (Spearman coefficient [ρ] and statistical significance [p] are reported in the graphics). Linear correlation was evaluated by using the regression test.

## Results

### Demographic and Clinical Findings

The study population included 102 consecutive COVID-19 subjects (median age and [IQR] of 66.5 [56.3–76.8], 63 male and 39 female) hospitalized from March 2020 to April 2020 and 47 HDs as the control group (median age and [IQR] of 61.0 [56.0–67.0], 22 male and 25 female). Demographic and clinical details of COVID-19 subjects are shown in **Table 1**.

**Table 1:** Demographic and clinical characteristics of COVID-19 subjects.

	COVID-19 subjects (n=102)	ARDS (n=60)	non-ARDS (n=42)	p value
Male/Female	63/39	43/17	20/22	0.0222*^a^*
Age, median (IRQ) years	66.5 (56.3–76.8)	72.0 (59.0–80.5)	62.0 (55.0–69.8)	0.0007*^b^*
Comorbidities				
Any	70	46	23	0.0308*^a^*
Hypertension	47	32	15	ns*^a^*
Cardiovascular	24	18	6	ns*^a^*
Pulmonary	23	14	2	0.0125*^a^*
Diabetes	19	12	7	ns*^a^*
Cancer	16	18	5	ns*^a^*
Renal	7	4	3	ns*^a^*
Symptoms				
Fever	90	55	35	–
Cough	54	36	18	–
Shortness of breath	47	37	10	–
Myalgia or arthralgia	25	11	14	–
Diarrhea	15	7	8	–
Anosmia and ageusia	3	1	2	–
Sputum production	1	1	0	–
Laboratory findings				
WBC (x109/L)	5.2 (4.0–7.7)	5.8 (4.5–8.5)	4.7 (3.5–6.3)	0.0032*^b^*
Neutrophils (x109/L)	3.7 (2.5–6.2)	4.3 (3.5–7.1)	3.2 (2.2–4.7)	0.0036*^b^*
Lymphocytes (x109/L)	0.9 (0.6–1.3)	0.9 (0.6–1.3)	0.9 (0.7–1.2)	ns*^b^*
NLR	4.5 (2.8–10.0)	6.1 (3.0–10.5)	3.6 (2.4–5.1)	0.0010*^b^*
CRP (mg/dl)	9.0 (3.4–18.9)	10.0 (4.5–20.7)	6.2 (1.8–15.9)	0.0436*^b^*
Ferritin (ng/ml)	546 (298.5–1139)	901 (345.5–2181)	420 (239.3–573.3)	0.0026*^b^*
LDH (U/L)	287 (242.5–381.5)	347 (276.3–424)	246 (213–295)	<0.0001*^b^*
D-dimer (µg/ml)	838.5 (340.8–1,629)	1328 (267.5–1,948)	598 (399–1,009)	ns*^b^*
IL-6 (pg/ml)	34.1 (10.8–80)	77.9 (33.1–80)	13.9 (2.7–27.5)	<0.0001*^b^*

ARDS, Acute distress respiratory syndrome; IQR, interquartile range; WBC, white blood cells; NLR, neutrophil/lymphocyte ratio; CRP, C-reactive protein; LDH, lactate dehydrogenase; IL-6, interleukine-6; ns, not significant.

a The 2-tailed χ2 test or Fisher’s exact test was used for comparing proportions between ARDS and non-ARDS groups.

b The nonparametric comparative Mann-Whitney test was used to compare medians between ARDS and non-ARDS groups.

According to chest CT scan findings, all COVID-19 subjects showed sign of interstitial pneumonia. On hospital admission, the most common symptoms were fever (88.2%), cough (52.9%), and dyspnea (46.1%). Sputum production was uncommon (1.0%). Concerning comorbidities, 63.7% had at least one coexisting illness and the prevalent were hypertension (46.1%), cardiovascular disease (23.5%), and pulmonary disease (22.5%) (**Table 1**).

According to clinical outcome, 58.8% of COVID-19 subjects had ARDS (ARDS group), while 41.2% of patients did not show signs of ARDS (non-ARDS group). Patients in the ARDS group were older than those in the non-ARDS group (p=0.0007). Moreover, the presence of any coexisting illness was more common among patients in the ARDS than in the non-ARDS group (38.7% vs 21.0%, p=0.0308) (**Table 1**). The median time (IQR) from hospital admission to the onset of ARDS was 6 (4-8) day.

On hospital admission, the ARDS group showed significantly higher levels of white blood cell (WBC) (p=0.0032) and neutrophils (p=0.0036) absolute counts, neutrophils/lymphocytes ratio (NLR) (p=0.0010), CRP (p=0.0436), ferritin (p=0.0026), LDH (p<0.0001), and IL-6 (p<0.0001) compared to non-ARDS group (**Table 1**). A higher median value of D-dimer was observed in the ARDS than the non-ARDS group, although not statistically significant (p=0.0844) (**Table 1**).

### Evaluation of sCD163 and sCD14 Plasmatic Levels

**Figure 2:**
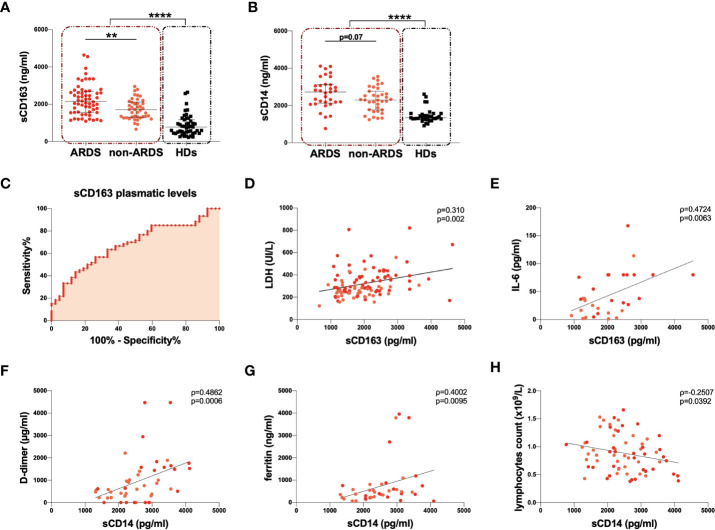
Evaluation of sCD163 and sCD14 plasmatic levels and correlations with clinical data. **(A)** sCD163 plasmatic levels were evaluated in 102 COVID-19 subjects (red dashed line box) and 47 healthy donors (HDs) (black dashed line box). The differences were evaluated using the nonparametric Mann-Whitney test. Moreover, sCD163 plasmatic levels were evaluated in 60 patients with acute respiratory distress syndrome (ARDS) (ARDS group) and 42 patients without ARDS (non-ARDS group). Differences were evaluated using the nonparametric Mann-Whitney test. Data are shown as median (lines) and interquartile ranges (whiskers). **(B)** sCD14 plasmatic levels were evaluated in 68 COVID-19 subjects (red dashed line box) and 35 HDs (black dashed line box). The differences were evaluated using the nonparametric Mann-Whitney test. Moreover, sCD14 plasmatic level was evaluated in 33 patients with ARDS (ARDS group) and 35 patients without ARDS (non-ARDS group) using the nonparametric Mann-Whitney test. Data are shown as median (lines) and interquartile ranges (whiskers). **(C)** ROC analysis was performed using sCD163 plasmatic levels after stratification of COVID-19 subjects according to the severity of the disease. The area under the curve is 0.6786 with p=0.0022. The cut-off of >2,023 pg/ml was identified with the Youden’s test and showed a sensitivity of 56.7% (CI: 44.1% to 68.4%) and a specificity of 73.8% (CI: 58.9% to 84.7%). CI: confidence interval. **(D)** Positive correlation between sCD163 and lactate dehydrogenase (LDH) plasmatic levels on 102 COVID-19 subjects. Linear correlation was evaluated by using the regression test, R^2^ = 0.099, p=0.001. Patients with ARDS are represented in red, while patients without ARDS in orange. **(E)** Positive correlation between sCD163 and IL-6 plasmatic levels on 32 COVID-19 subjects. Linear correlation was evaluated by using the regression test, R^2^ = 0.242, p=0.004. Patients with ARDS are represented in red, while patients without ARDS in orange. **(F)** Positive correlation between sCD14 level and D-dimer plasmatic levels on 46 COVID-19 subjects. Linear correlation was evaluated by using the regression test, R^2^ = 0174 p=0.004. Patients with ARDS are represented in red, while patients without ARDS in orange. **(G)** Positive correlation between sCD14 and ferritin plasmatic levels on 41 COVID-19 subjects. Linear correlation was evaluated by using the regression test, R^2^ = 0.101 p=0.043. Patients with ARDS are represented in red, while patients without ARDS in orange. **(H)** Negative correlation between sCD14 plasmatic levels and absolute count of lymphocytes on 68 COVID-19 subjects. Linear correlation was evaluated by using the regression test, R^2^ = 0.058 p=0.048. Patients with ARDS are represented in red, while patients without ARDS in orange. All correlations were performed using Spearman test. Spearman coefficient (ρ) and statistical significance (p) are reported in the graphics. ****p<0.0001; **0.01<p<0.001.

sCD163 plasmatic levels were compared between 102 COVID-19 subjects and 47 HDs. On hospital admission, COVID-19 subjects showed significantly higher sCD163 plasmatic levels compared to HDs (p<0.0001) (**Table 2**, **Figure 2A**).

**Table 2:** sCD163 and sCD14 plasmatic levels and immunophenotyping data.

	COVID-19 subjects (n=45)	HDs (n=19)	ARDS (n=17)	non-ARDS (n=28)	p value*^a^*	p value*^b^*	p value*^c^*
sCD163 (ng/ml)	1,943 (1,373–2,474)	776.7 (457.6–1169)	2,146 (1,564–2,680)	1,700 (1,300–2,117)	<0.0001	<0.0001	<0.0001
sCD14 (ng/ml)	2,389 (2,062–3,024)	1,341 (1,273–1,529)	2,725 (2,128–3,125)	2,298 (1,769–2,766)	<0.0001	<0.0001	<0.0001
Non-classical monocytes (%)	7.2 (5.3–9.9)	11.0 (7.7–14.9)	6.1 (3.1–7.0)	9.0 (6.0–11.4)	<0.0001	<0.0001	ns
Intermediate monocytes (%)	5.5 (3.7–8.0)	8.9 (5.1–10.0)	4.1 (2.4–7.1)	5.8 (4.1–8.3)	0.0494	0.0284	ns
Classical monocytes (%)	43.0 (30.2–52.8)	39.1 (29.5–47.9)	34.3 (25.6–53.3)	44.7 (32.5–52.3)	ns	ns	ns
SlanDCs (%)	2.4 (1.0–4.4)	21.9 (17.7–30.9)	2.4 (1.7–7.4)	2.4 (0.2–4.1)	<0.0001	<0.0001	<0.0001
mDCs (%)	22.2 (13.8–32.1)	42.0 (36.7–53.5)	20.0 (10.1–25.6)	27.7 (15.2–40.7)	<0.0001	<0.0001	0.0041
pDCs (%)	32.0 (22.1–43.0)	43.3 (37.7–47.1)	27.1 (22.1–32.2)	39.4 (22.5–46.0)	0.0005	0.0002	ns

HDs, healthy donors; ARDS, Acute distress respiratory syndrome; mDCs, myeloid dendritic cells; pDCs, plasmacytoid dendritic cells; ns, not significant.

a The nonparametric comparative Kruskal-Wallis test was used for comparing medians of ARDS and non-ARDS groups and HDs.

b The Dunn’s multiple comparison post-test was used for comparing medians of ARDS group with HDs.

c The Dunn’s multiple comparison post-test was used for comparing medians of non-ARDS group with HDs.

The ARDS group showed significantly higher sCD163 plasmatic levels compared to the non-ARDS group (p=0.002) (**Table 2**, **Figure 2A**). Both the ARDS and non-ARDS groups showed significantly higher sCD163 plasmatic levels compared to HDs (p<0.0001 and p<0.0001, respectively) (**Table 2**). A negative correlation between the number of days from hospital admission to the onset of ARDS and sCD163 plasmatic levels was found (ρ=-0.315, p=0.013) (**Supplementary Figure 1C**). Given the evidence of increased sCD163 plasmatic levels associated with the severity of the disease, the Receiving Operator Characteristic (ROC) analysis was performed, and a cut-off value of 2032 ng/ml was found, indicating that a baseline sCD163 plasmatic level greater than 2032 ng/ml was predictive of a more severe disease (**Figure 2C**).

The evaluation of sCD163 plasmatic levels in COVID-19 subjects stratified according to WHO guidelines, into three groups (critical, severe and mild) showed a higher sCD163 plasmatic level in the critical group compared to the mild one (p=0.004). The same trend was observed in the severe group compared to the mild one, although not statistically significant. No significant differences were observed between critical and severe groups (**Supplementary Table 1**, **Supplementary Figure 1A**).

Considering all COVID-19 subjects, we observed a positive correlation between sCD163 and LDH plasmatic levels (ρ=0.310, p=0.002) (**Figure 2D**) and between sCD163 and IL-6 plasmatic levels (ρ=0.472, p=0.006) (**Figure 2E**). No further correlations were found between sCD163 plasmatic levels and other clinical parameters. No association between sCD163 plasmatic levels and age of the COVID-19 subjects was observed nor differences between males and females.

sCD14 plasmatic levels were compared between 68 COVID-19 subjects and 35 HDs. sCD14 plasmatic levels were significantly higher in COVID-19 subjects compared to HDs (p<0.0001) (**Table 2**, **Figure 2B**). A slight increase in the ARDS compared to the non-ARDS group was observed (p=0.07) (**Table 2**, **Figure 2B**). Both the ARDS and non-ARDS groups showed higher sCD14 plasmatic levels compared to HDs (p<0.0001 and p<0.0001, respectively). No correlation between the number of days from hospital admission to the onset of ARDS and sCD14 plasmatic levels was found.

The further evaluation of sCD14 plasmatic levels in COVID-19 subjects stratified into three groups: critical, severe and mild, showed a higher sCD14 plasmatic level in the critical and severe groups compared to the mild one, although not statistically significant. No significant differences were observed between the critical and severe groups (**Supplementary Table 1**, **Supplementary Figure 1B**).

We observed a strong positive correlation between sCD14 and D-dimer plasmatic levels (ρ=0.486, p=0.0006) (**Figure 2F**) and between sCD14 and ferritin plasmatic levels (ρ=0.400, p=0.010) (**Figure 2G**). Finally, a negative correlation between sCD14 plasmatic levels and lymphocyte absolute counts (ρ=-0.251, p=0.039) (**Figure 2H**) was documented. No association between sCD14 plasmatic levels and age of the COVID-19 subjects was observed nor differences between males and females.

### Monocyte and DC Subsets

**Figure 3:**
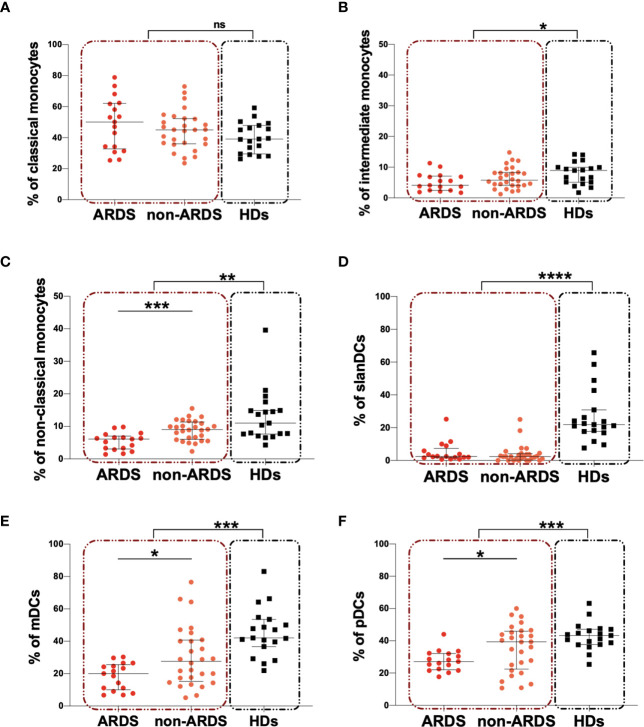
Peripheral blood monocyte and DC subsets in COVID-19 subjects compared to HDs and according to the severity of the disease. **(A)** The percentage of classical monocyte was evaluated in 45 COVID-19 subjects (red dashed line box) and 19 HDs (black dashed line box). The differences were evaluated using the nonparametric Mann-Whitney test. Moreover, the percentages of classical monocyte were evaluated in 17 patients with ARDS (ARDS group) and in 28 patients without ARDS (non-ARDS group) using the nonparametric Mann-Whitney test. Data are shown as median (lines) and interquartile ranges (whiskers). **(B)** The percentage of intermediate monocyte was evaluated in 45 COVID-19 subjects (red dashed line box) and 19 HDs (black dashed line box). The differences were evaluated using the nonparametric Mann-Whitney test. Moreover, the percentages of intermediate monocyte were evaluated in 17 patients with ARDS (ARDS group) and in 28 patients without ARDS (non-ARDS group) using the nonparametric Mann-Whitney test. Data are shown as median (lines) and interquartile ranges (whiskers). **(C)** The percentage of non-classical monocyte was evaluated in 45 COVID-19 subjects (red dashed line box) and 19 HDs (black dashed line box). The differences were evaluated using the nonparametric Mann-Whitney test. Moreover, the percentages of non-classical monocyte were evaluated in 17 patients with ARDS (ARDS group) and in 28 patients without ARDS (non-ARDS group) using the nonparametric Mann-Whitney test. Data are shown as median (lines) and interquartile ranges (whiskers). **(D)** The percentage of slanDCs was evaluated in 45 COVID-19 subjects (red dashed line box) and 19 HDs (black dashed line box). The differences were evaluated using the nonparametric Mann-Whitney test. Moreover, the percentages of slanDCs were evaluated in 17 patients with ARDS (ARDS group) and in 28 patients without ARDS (non-ARDS group) using the nonparametric Mann-Whitney test. Data are shown as median (lines) and interquartile ranges (whiskers). **(E)** The percentage of mDCs was evaluated in 45 COVID-19 subjects (red dashed line box) and 19 HDs (black dashed line box). The differences were evaluated using the nonparametric Mann-Whitney test. Moreover, the percentages of mDCs were evaluated in 17 patients with ARDS (ARDS group) and in 28 patients without ARDS (non-ARDS group) using the nonparametric Mann-Whitney test. Data are shown as median (lines) and interquartile ranges (whiskers). **(F)** The percentage of pDCs was evaluated in 45 COVID-19 subjects (red dashed line box) and 19 HDs (black dashed line box). The differences were evaluated using the nonparametric Mann-Whitney test. Moreover, the percentages of pDCs were evaluated in 17 patients with ARDS (ARDS group) and in 28 patients without ARDS (non-ARDS group) using the nonparametric Mann-Whitney test. Data are shown as median (lines) and interquartile ranges (whiskers). mDCs, myeloid dendritic cells; pDCs, plasmacytoid dendritic cells; ns, not significant. *0.05<p<0.01; **0.01<p<0.001; ***p<0.001; ****p>0.0001.

For 45 COVID-19 subjects and 19 HDs peripheral blood monocyte and DC subsets were characterized. The median percentages and the IQR of peripheral blood monocytes and DCs are summarized in **Table 2**. On hospital admission, COVID-19 subjects showed a significantly lower percentage of intermediate and non-classical monocytes compared to HDs (p=0.045 and p=0.001, respectively) (**Table 2**). No statistically significant difference was observed in the percentage of classical monocytes, although the median value was higher in the COVID-19 subjects compared to HDs (**Table 2**). A significantly lower percentage of non-classical monocytes was observed in the ARDS compared to the non-ARDS group (p=0.0006) (**Figure 3C**). No statistically differences were found in the percentage of both classical and intermediate monocytes, after comparing the ARDS and non-ARDS groups (**Figures 3A, B**). Only the ARDS group showed a significantly lower percentage of intermediate and non-classical monocytes compared to HDs (p=0.0284 and p<0.0001, respectively) (**Table 2**) (**Figures 3B, C**, respectively).

Finally, we observed a significantly lower percentage of slanDCs, mDCs and pDCs in COVID-19 subjects compared to HDs (p<0.0001, p=0.0006, and p=0.026, respectively) (**Table 2**). No differences for slanDCs were observed between the ARDS and non-ARDS groups (**Figure 3D**). Intriguingly, a lower percentage of mDCs and pDCs in the ARDS compared to non-ARDS group was observed (p=0.0346 and p=0.0492, respectively) (**Figures 3E, F**, respectively). Both the ARDS and non-ARDS groups showed lower percentage of slanDCs and mDCs compared to HDs (for slanDCs p<0.0001 and p<0.0001, respectively; for mDCs: p<0.0001 and p=0.0041, respectively) (**Figures 3D, E**, respectively) (**Table 2**). For pDCs, only the ARDS group showed a significantly lower percentage in respect to HDs (p=0.0002) (**Table 2**) (**Figure 3F**).

In a further evaluation, after stratifying patients according to WHO guidelines, a significant depletion of non-classical monocytes in critical group compared to mild one (p=0.004) was observed. The same trend was observed in severe group compared to the mild one, although not statistically significant. No significantly differences were observed between critical and severe groups (**Supplementary Table 1**, **Supplementary Figure 1F**). For intermediate and classical monocytes and DC subsets no significant differences were observed after comparing the three groups (**Supplementary Table 1**, **Supplementary Figure 1**).

### Correlation Between Soluble Marker of Monocytes/Macrophages Activation and Immunophenotyping Analysis Data in COVID-19 Subjects

**Figure 4:**
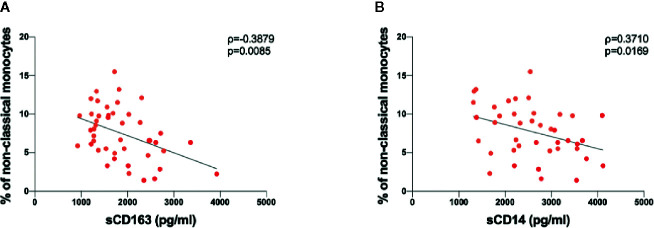
Correlations between sCD163 and sCD14 plasmatic levels and immunophenotyping analysis data performed in COVID-19 subjects. **(A)** Negative correlation between sCD163 plasmatic levels and percentages of non-classical monocytes. Linear correlation was evaluated by using the regression test, R^2^ = 0.179 p=0.004. Patients with acute respiratory distress syndrome (ARDS) are represented in red, while patients without ARDS in orange. **(B)** Positive correlation between sCD14 plasmatic levels and percentages of non-classical monocytes. Linear correlation was evaluated by using the regression test, R^2^ = 0.134 p=0.019. Patients with ARDS are represented in red, while patients without ARDS in orange. All correlations were performed using Spearman test. Spearman coefficient (ρ) and statistical significance (p) are reported in the graphics.

Spearman rank correlations were used to explore associations between soluble plasma biomarkers and monocyte and DC subsets. We found a negative correlation between sCD163 plasmatic levels and the percentage of non-classical monocytes (ρ=-0.388, p=0.009) (**Figure 4A**). Finally, a negative correlation was found between sCD14 plasmatic levels and the percentage of non-classical monocytes (ρ=-0.371, p=0.017) (**Figure 4B**).

## Discussion

In the present study, we assessed biomarkers of monocyte/macrophage activation by evaluating sCD163 and sCD14 plasmatic levels and characterized peripheral blood monocyte and DC subsets in patients with COVID-19 pneumonia at the time of hospital admission. Moreover, we analyzed the association between biomarkers of monocyte/macrophage activation and clinical, laboratory and immunophenotyping data.

Although overlapping, sCD163 and sCD14 may represent distinct biological pathways of monocyte/macrophage lineage activation (11, 17, 18). Indeed, sCD163 and sCD14 are associated with the innate immune response and are produced following the infection of target cells, such as monocytes and tissue macrophages. CD163 is an important surface marker expressed on monocyte/macrophage lineage cells and is shed in the soluble form (sCD163) during inflammatory events to quench the cascade of inflammation. Because of its prevalent expression on the monocytic lineage, sCD163 can be considered a specific marker of tissue macrophages and monocyte activation (11). CD14 is a co-receptor for lipopolysaccharide, and monocyte release of sCD14 is mediated by the translocation of microbial products from the gut into the systemic circulation (37, 38).

Increased sCD163 plasmatic levels have been found in several infections (HIV, HIV/CMV co-infection, hepatitis, sepsis, tuberculosis) (39–42). Moreover, high sCD14 plasmatic levels were reported in patients with ARDS, bacterial pneumonia and diffuse interstitial lung diseases (16). Data regarding sCD163 and sCD14 plasmatic levels in patients with COVID-19 pneumonia are still limited, and their potential use as prognostic markers is under investigation in a few reports from the scientific literature (43).

The first main result of our study was that COVID-19 subjects showed higher baseline sCD163 and sCD14 plasmatic levels compared to HDs, indicating an early activation of the monocytic/macrophage system, which can be documented on hospital admission. This finding is in line with a recent report by Gómez-Rial et al. that showed higher sCD163 and sCD14 plasmatic levels in patients with COVID-19 pneumonia compared to controls (43). In our study, after stratifying COVID-19 subjects according to the development of ARDS, we observed higher baseline sCD163 and sCD14 plasmatic levels in the ARDS compared to the non-ARDS group. Similar results were obtained after stratifying patients into three groups: critical, severe and mild, according to WHO guidelines. These data underlined the presence of a higher immune activation of the monocytic lineage in patients who developed ARDS (or had a worst outcome) compared to patients who did not. Specifically, considering that monocyte activation soluble markers are increased at the moment of hospitalization, they could represent a valuable predictive marker of severe disease in COVID-19 patients. Indeed, we observed a negative correlation between sCD163 plasmatic levels and the number of days from hospital admission to the onset of ARDS. This could indicate that higher sCD163 plasmatic levels are associated to a faster progression to ARDS. Furthermore, we found a cut-off value for sCD163 plasmatic levels, which was associated with the severity of the disease. Patients with baseline plasmatic levels above this cut-off were at increased risk of developing ARDS.

We found a positive correlation between sCD163 and LDH plasmatic levels, underlining a possible link between monocyte/macrophage activation and tissue damage. Moreover, we observed a positive correlation between sCD163 and IL-6 plasmatic levels. Interestingly, we found a positive correlation between sCD14 and D-dimer plasmatic levels indicating a possible link between monocyte activation and hypercoagulability, which is one of the major complications in COVID-19 pneumonia. The positive correlation between sCD14 and ferritin plasmatic levels has been already reported by other authors, underlining the link between high ferritin level and monocyte activation in peripheral blood (44).

The second main result of our study was the significantly lower percentage of proinflammatory monocytes, such as non-classical and intermediate monocytes, in patients with COVID-19 pneumonia compared to HDs. Moreover, the percentages of proinflammatory monocytes were lower in the ARDS compared to the non-ARDS group as well as in the critical and severe groups compared to the mild one. These results disagree with data published by Gibellini et al., in which an enrichment of proinflammatory monocytes in peripheral blood of COVID-19 subjects was reported (45). Conversely, our data are in line with other reports in which a depletion of proinflammatory monocytes in COVID-19 patients was described and a major depletion of these cells was associated with a more severe clinical outcome (46, 47). This controversial situation could be due to a different time of sample collection corresponding to a different phase of COVID-19. Unfortunately, the authors did not specify when the analyzed samples were taken, without indicating the number of days from symptom onset or hospital admission to sample collection).

A prior review article has been published, describing the accumulation of monocyte/macrophage cells in the lungs, which are likely sources of proinflammatory cytokines and chemokines associated with fatal disease induced by human coronavirus infections, including COVID-19 (48). Based on these findings, it is possible that the decrease in proinflammatory monocytes could be due to migration into the lung tissue instead of bone marrow depletion. This hypothesis is corroborated by recently data by Sanchez-Cerrillo et al., in which the comparison between the characterization of monocyte and DC subsets in peripheral blood and bronchoalveolar lavage fluid samples of COVID-19 subjects showed as the depletion of proinflammatory monocyte and DC peripheral blood cells was associated with a selective recruitment in the lungs during the development of ARDS (46).

In COVID-19 subjects we observed a decrease in the percentages of slanDCs, an additional population of myeloid cells in the blood (28). SlanDCs represent a subset of atypical monocytes (20). Functionally, blood slanDCs have been described as potent proinflammatory cells based on their capacity to produce large amounts of TNF-α and IL-12 upon stimulation with toll-like receptor (TLR) ligands (28, 49). Moreover, it is well known that slanDCs are located in lymphoid and peripheral tissues, especially during inflammatory conditions (28) and they can be recruited in the lungs during COVID-19 pneumonia.

Finally, as reported by other authors, we observed a marked reduction in the percentage of mDCs and pDCs in the peripheral blood of COVID-19 subjects and the depletion of these subsets was more evident in patients with a worst outcome (46, 47). This phenomenon could be due to recruitment in the lungs, similarly to what has been described for Influenza H1N1 infection (30). Indeed, pDCs are specialized in secreting high amount of type 1 IFNs, which serve as antiviral molecule and initiate antiviral adaptive immune responses, while mDCs function as peripheral sentinels by transmitting antigen derived signals to draining lymph nodes and secrete high levels of IL-12 and are key players in amplifying adaptive immune responses (50).

The negative correlation between sCD163 plasmatic levels and the percentage of atypical monocytes is in line with previous reports that showed that CD163 is more expressed in classical compared to intermediate monocytes, and not expressed in non-classical monocytes, considering both protein and gene expression (51). In line with other authors (52), we observed a negative correlation between the percentage of non-classical monocytes and sCD14 plasmatic levels.

Limitations of our study include the lack of the evaluation of sCD14 plasmatic levels and immunophenotyping analysis on the whole study population. Moreover, in our study, we did not explore cellular infiltrates in lungs.

Overall, this study supports the growing body of evidences that monocytes and macrophages could play a paramount role in the pathogenesis of COVID-19 pneumonia, contributing to tissue inflammation. We speculate that monitoring these immune activation markers at baseline has prognostic significance throughout the course of COVID-19 pneumonia. Specifically, given the correlation between sCD163 and sCD14 plasmatic levels and other inflammatory biomarker, we speculate that the alteration of sCD163 and sCD14 signaling systems play a role in the pathogenesis of COVID-19, although the underlying mechanisms should be further explored.

In summary, our findings support the hypothesis that the depletion of peripheral blood pro-inflammatory monocytes, mDCs and pDCs could be due to recruitment in the lungs during COVID-19. In an early stage of the disease, the assessment of sCD163 plasmatic levels could have clinical utility in predicting the severity of COVID-19 pneumonia.

## Data Availability Statement

The datasets presented in this study can be found in online repositories. The names of the repository/repositories and accession number(s) can be found below: The cytofluorimetric source data underlying **Figure 1** is available at the http://doi.org/10.5281/zenodo.4404888.

## Ethics Statement

The studies involving human participants were reviewed and approved by Ethics Committee of Policlinico Umberto I, Sapienza University of Rome (protocol number 298/2020) and the Ethic Committee Lazio 2 (protocol number 0080757/2020). The patients/participants provided their written informed consent to participate in this study.

## Author Contributions

MZ and ML: designed the study. MZ: performed laboratory testing, analyzed data, performed statistical analysis, and wrote the manuscript. PN and VP: assisted in designing the study, performed laboratory testing, and analyzed data. MI and ML: discussed results and critically revised the manuscript. ACa, PP, PZ, MR, KB, GS, CB, ACu, and CA: provided clinical samples and clinical data. MC, CM, and ML discussed result, read and revised the manuscript. All authors contributed to the article and approved the submitted version.

## Conflict of Interest

The authors declare that the research was conducted in the absence of any commercial or financial relationships that could be construed as a potential conflict of interest.
