# Supplementary material for: Increased sCD163 and sCD14 Plasmatic Levels and Depletion of Peripheral Blood Pro-Inflammatory Monocytes, Myeloid and Plasmacytoid Dendritic Cells in Patients With Severe COVID-19 Pneumonia
Source: Front Immunol. 2021 Feb 26;12:627548. doi: 10.3389/fimmu.2021.627548 (PMC7993197; doi:10.3389/fimmu.2021.627548)
Supplement: Supplementary file 1 [file DataSheet_1.pdf]

Supplementary Material

Supplementary table 1. sCD163 and sCD14 plasmatic levels and immunophenotyping data in COVID-19 subjects classified into three groups: critical, severe and mild (according to the WHO classification)

	critical (n=18)	severe (n=44)	mild (n=40)	p value[1]	p value[2]	p value[3]	p value[4]
sCD163 (ng/ml)	2404 (1892-3298)	2040 (1546-2602)	1700 (1314-2086)	0.003	ns	0.004	ns
sCD14 (ng/ml)	2841 (2149-3746)	2774 (2080-3067)	2292 (1749-2658)	ns	ns	ns	ns
Non-classical monocytes (%)	3.3 (1.6-6.6)	6.2 (4.0-8.3)	9.0 (6.0-11.4)	0.002	ns	0.005	ns
Intermediate monocytes (%)	3.7 (2.4-7.3)	5.4 (3.4-7.0)	5.8 (4.1-8.3)	ns	ns	ns	ns
Classical monocytes (%)	47.0 (31.4-58.1)	53.3 (32.2-63.8)	44.7 (32.5-52.3)	ns	ns	ns	ns
SlanDCs (%)	5.9 (1.9-11.5)	2.4 (0.9-3.7)	2.4 (0.2-4.1)	ns	ns	ns	ns
mDCs (%)	18.5 (9.1-24.6)	20.8 (10.3-27.3)	27.7 (15.2-40.7)	ns	ns	ns	ns
pDCs (%)	28.2 (22.5-33.6)	25.8 (21.3-30.9)	39.4 (22.5-46.0)	ns	ns	ns	ns

mDCs: myeloid dendritic cells; pDCs: plasmacytoid dendritic cells. [1]: The nonparametric comparative Kruskal-Wallis test was used for comparing medians of critical, severe and mild groups. [2]: The Dunn's multiple comparison post-test was used for comparing medians of critical and severe groups. [3]: The Dunn's multiple comparison post-test was used for comparing medians of critical and mild groups. [4]: The Dunn's multiple comparison post-test was used for comparing medians of severe and mild groups.

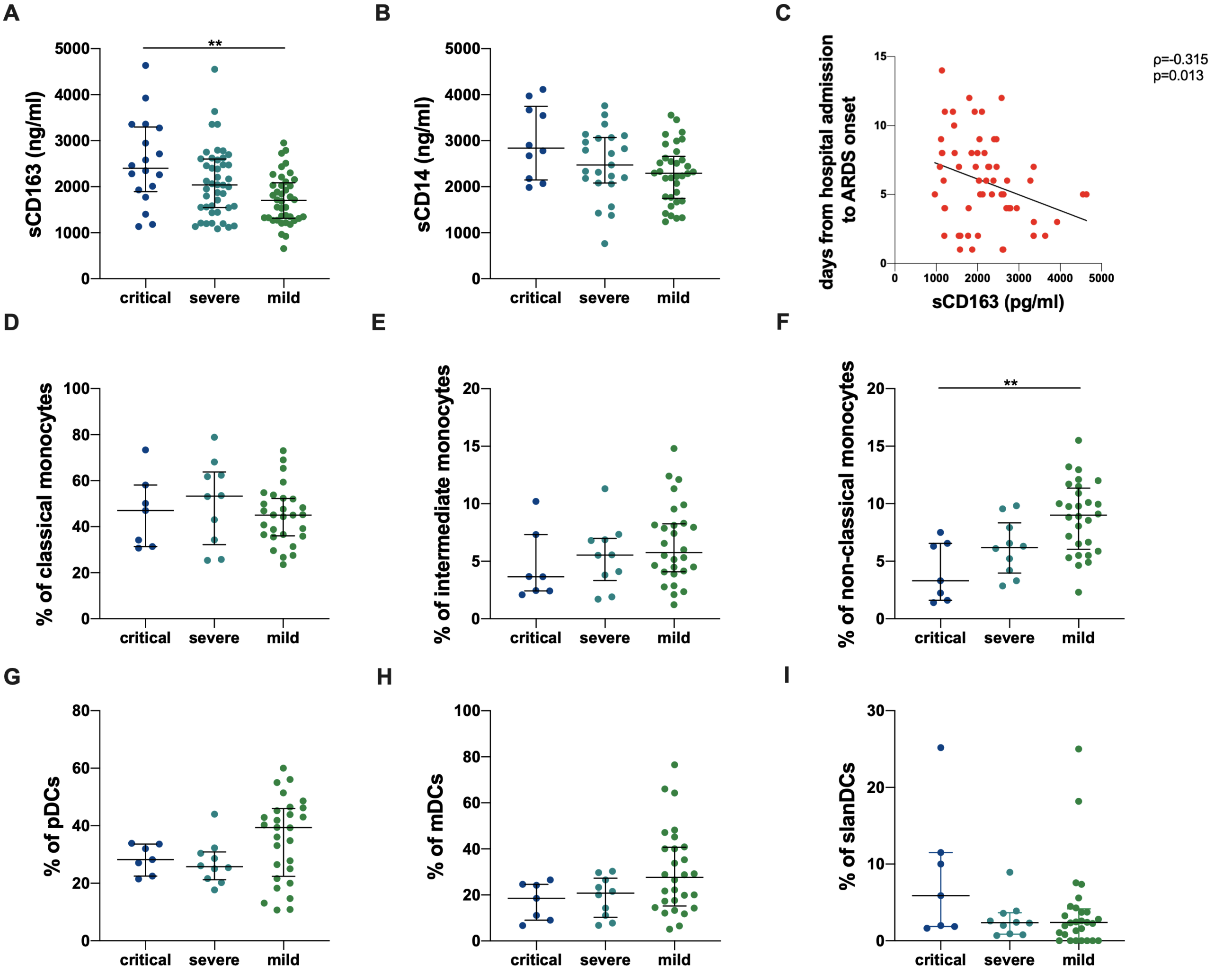

Supplementary Figure 1. Evaluation of sCD163 and sCD14 plasmatic levels and peripheral blood monocyte and DC subsets in COVID-19 subjects classified into three groups: critical, severe and mild (according to the WHO classification). (**A**) sCD163 plasmatic levels were evaluated in 18 patients with severe, 40 patients with critical and 44 patients with mild COVID-19. The differences were evaluated using the nonparametric Kruskal-Wallis test and Dunn's multiple comparison post-test for comparing medians of groups. Data are shown as median (lines) and interquartile ranges (whiskers). (**B**) sCD14 plasmatic levels were evaluated in 10 patients with severe, 23 patients with critical and 34 patients with mild COVID-19. The differences were evaluated using the nonparametric Kruskal-Wallis test and Dunn's multiple comparison post-test for comparing medians of groups. Data are shown as median (lines) and interquartile ranges (whiskers). (**C**) Negative correlation between sCD14 plasmatic levels and days from hospital admission to ARDS onset on 102 COVID-19 subjects. Correlation was performed using Spearman test. Spearman coefficient (ρ) and statistical significance (p) are reported in the graphics. Linear correlation was evaluated by using the regression test, R^2=0.093 p=0.017. (**D**) The percentage of classical monocyte was evaluated in 7 patients with severe, 10 patients with critical and 28 patients with mild COVID-19. The differences were evaluated using the nonparametric Kruskal-Wallis test and Dunn's multiple comparison post-test for comparing medians of groups. Data are shown as median (lines) and interquartile ranges

(whiskers). (**E**) The percentage of intermediate monocyte was evaluated in 7 patients with severe, 10 patients with critical and 28 patients with mild COVID-19. The differences were evaluated using the nonparametric Kruskal-Wallis test and Dunn's multiple comparison post-test for comparing medians of groups. Data are shown as median (lines) and interquartile ranges (whiskers). (**F**) The percentage of non-classical monocyte was evaluated in 7 patients with severe, 10 patients with critical and 28 patients with mild COVID-19. The differences were evaluated using the nonparametric Kruskal-Wallis test and Dunn's multiple comparison post-test for comparing medians of groups. Data are shown as median (lines) and interquartile ranges (whiskers). (**G**) The percentage of pDCs was evaluated in 7 patients with severe, 10 patients with critical and 28 patients with mild COVID-19. The differences were evaluated using the nonparametric Kruskal-Wallis test and Dunn's multiple comparison post-test for comparing medians of groups. Data are shown as median (lines) and interquartile ranges (whiskers). (**H**) The percentage of mDCs was evaluated in 7 patients with severe, 10 patients with critical and 28 patients with mild COVID-19. The differences were evaluated using the nonparametric Kruskal-Wallis test and Dunn's multiple comparison post-test for comparing medians of groups. Data are shown as median (lines) and interquartile ranges (whiskers). (**I**) The percentage of slanDCs was evaluated in 7 patients with severe, 10 patients with critical and 28 patients with mild COVID-19. The differences were evaluated using the nonparametric Kruskal-Wallis test and Dunn's multiple comparison post-test for comparing medians of groups. Data are shown as median (lines) and interquartile ranges (whiskers).

**: $0.01<p<0.001$
